# Case Report: Chronic Adaptive Deep Brain Stimulation Personalizing Therapy Based on Parkinsonian State

**DOI:** 10.3389/fnhum.2021.702961

**Published:** 2021-08-13

**Authors:** Asuka Nakajima, Yasushi Shimo, Atsuhito Fuse, Joji Tokugawa, Makoto Hishii, Hirokazu Iwamuro, Atsushi Umemura, Nobutaka Hattori

**Affiliations:** ^1^Department of Neurology, Juntendo University Nerima Hospital, Tokyo, Japan; ^2^Department of Research and Therapeutics for Movement Disorders, School of Medicine, Juntendo University, Tokyo, Japan; ^3^Department of Neurosurgery, Juntendo University Nerima Hospital, Tokyo, Japan; ^4^Department of Neurosurgery, School of Medicine, Juntendo University, Tokyo, Japan; ^5^Department of Neurology, School of Medicine, Juntendo University, Tokyo, Japan

**Keywords:** Parkinson's disease, adaptive deep brain stimulation, beta oscillation, local field potential, physiological biomarker

## Abstract

We describe the case of a 51-year-old man with Parkinson's disease (PD) presenting with motor fluctuations, who received bilateral subthalamic deep brain stimulation (DBS) with an adaptive DBS (aDBS) device, Percept™ PC (Medtronic, Inc. , Minneapolis, MN). This device can deliver electrical stimulations based on fluctuations of neural oscillations of the local field potential (LFP) at the target structure. We observed that the LFP fluctuations were less evident inside the hospital than outside, while the stimulation successfully adapted to beta oscillation fluctuations during the aDBS phase without any stimulation-induced side effects. Thus, this new device facilitates condition-dependent stimulation; this new stimulation method is feasible and provides new insights into the pathophysiological mechanisms of PD.

## Introduction

Deep brain stimulation (DBS) is widely used to treat advanced Parkinson's disease (PD). To adapt to the stimulation parameters, adaptive DBS (aDBS) uses the local field potential (LFP) of the target structure recorded through the implanted electrodes that deliver stimulation (Little and Brown, 2014). An abnormal LFP oscillation (beta band oscillation [13–30 Hz]) is observed in the subthalamic nucleus (STN) and is modulated by levodopa (Kuhn et al., 2006) or movement (Androulidakis et al., 2008). Furthermore, the power of these beta band oscillations is correlated with the akinesia and rigidity of parkinsonism (Kuhn et al., 2006) and is considered the most promising biomarker for aDBS in PD (Bouthour et al., 2019). The aDBS system can change the current depending on the strength of the beta band oscillation, and can, therefore, overcome conventional DBS (cDBS) therapy limitations, including stimulation-induced long term side effects, such as dyskinesia (Arlotti et al., 2018) or speech deterioration (Little et al., 2016). However, to date, no studies have assessed oscillations based on the patients' conditions, such as inside or outside the hospital, for a long period. Herein, we report the differences in beta band oscillations based on the patient's circumstances.

## Case Description

**Figure 1:**
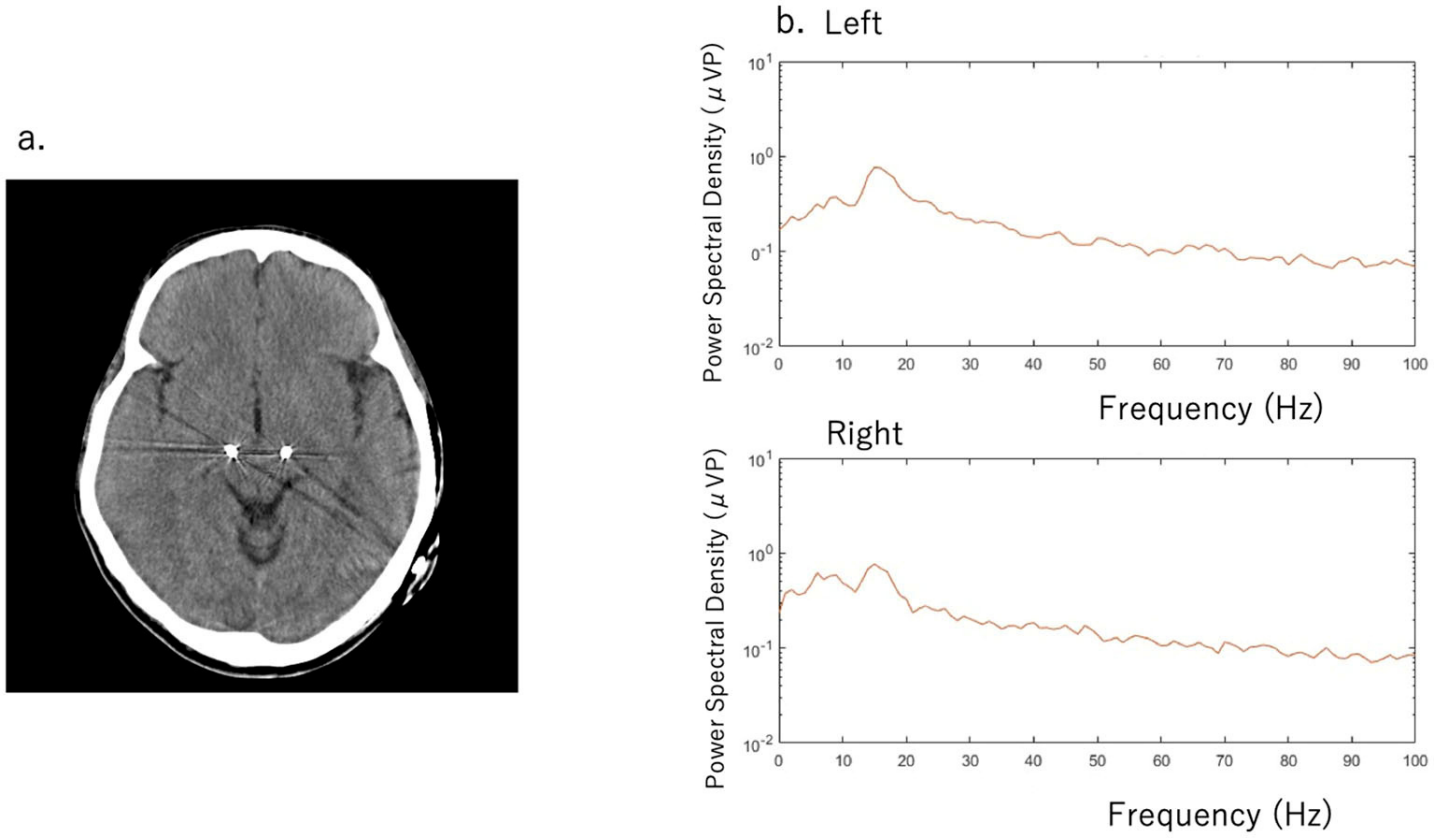
**(a)** Computed tomography scan with deep brain stimulation (DBS) electrodes **(b)** Beta band oscillation was detected in the left subthalamic nucleus (STN) (16.6 Hz with 1.20 μVp) and in the right STN (1.72 μVp) during the med-off/stim-off phase. The Movement Disorder Society revision of the United Parkinson's Rating Scale (MDS-UPDRS) score was 27 points.

**Figure 2:**
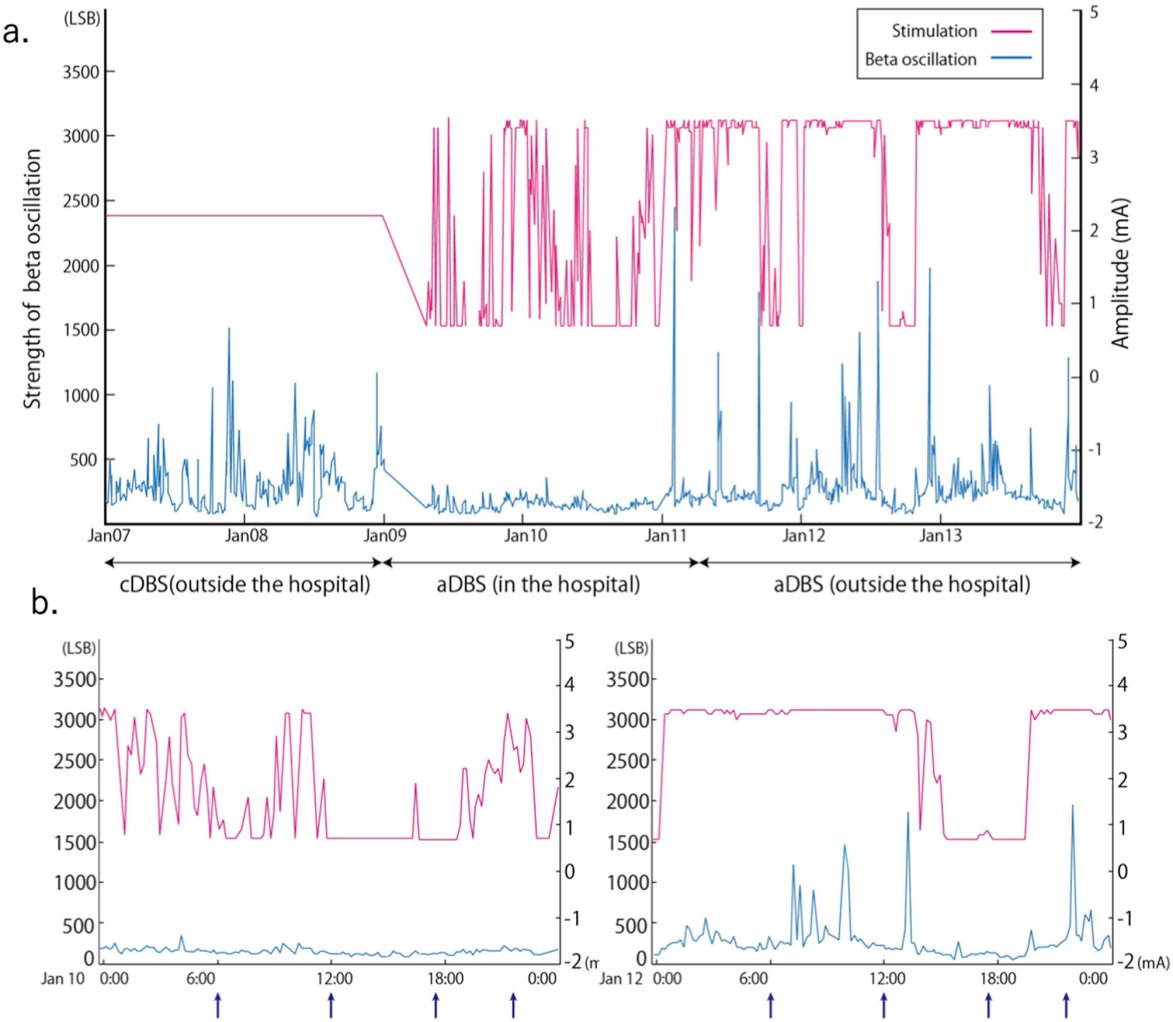
Stimulation amplitude of adaptive deep brain stimulation (aDBS) and fluctuations in the beta band (16.60 ± 2.5 Hz) oscillation of the local field potential (LFP) over time for a long period **(a)** and a short period **(b)**. Arrow in **(b)** indicates the timing of levodopa 100 mg /carbidopa 25 mg intake. Conventional DBS (cDBS) (outside the hospital): Stimulation was set at 2.2 mA. aDBS (inside and outside the hospital): Upper stimulation was set at 3.5 mA and lower at 0.7 mA. Strength of beta oscillation = Vin (v) * Gain * 16 (LSB) / 1.2 (v) Vin: input voltage to the analog to digital convertor, Gain = 250, LSB of an analog-to-digital convertor is 16 least significant bits, The analog-to-digital convertor has a voltage range of 1.2 v.

**Figure 3:**
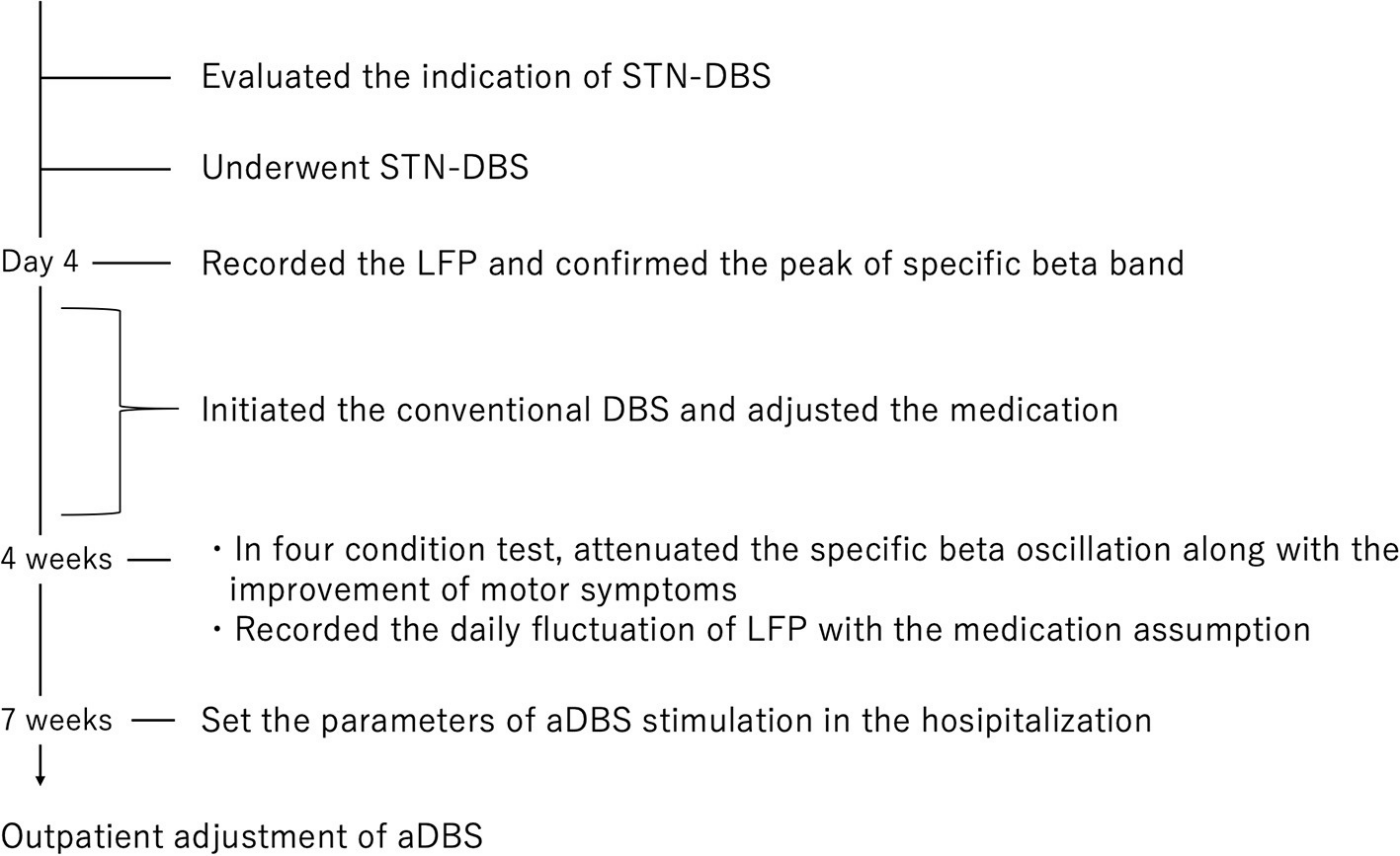
Clinical course of the patient.

A 51-year-old man with a 16-year history of PD was admitted to our hospital with an indication for STN-DBS. He presented with severe motor fluctuations and off symptoms for more than 2 h a day. His routine medications included levodopa/carbidopa (800 mg, 8 times), pramipexole (1.5 mg), zonisamide (25 mg), and istradefylline (20 mg). He showed no cognitive decline according to the Japanese version of the Montreal Cognitive Assessment (26 points) and Frontal Assessment Battery (17 points) and no psychiatric symptoms (Neuropsychiatric Inventory: 10 points, Beck depression Inventory: 29 points). His Movement Disorder Society revision of the Unified Parkinson's Disease Rating Scale (MDS-UPDRS) motor score was 38 in the off-state (withdrawal of anti-parkinsonian drugs for >12 h) and four in the on-state (1 h following the administration of 1.5 times higher than usual morning levodopa dose; 150 mg/50 mg of levodopa/decarboxylase inhibitor following the drug-off phase). After these evaluations, he consented to undergo STN-DBS with Percept PC (Medtronic, Inc., Minneapolis, MN) (Figure 1a). Four days after surgery, we recorded the bipolar LFP activity from contacts 1–3 and 9–11 of the electrodes (contacts 0 and 8 were the most ventral contacts of the left-sided and right-sided electrodes, respectively) in the STN. The contact selected for stimulation was situated between the bipolar contact selected for recording (contact 2 or 10). We could confirm the specific beta oscillation (16.6 Hz, 1.07 μVp) in the off-state before applying the stimulation. Following a manual assessment of rigidity, finger tapping, and pronation-supination movements with DBS off, the current was increased by 1.0-mA increments starting from 0 mA to the point of reducing parkinsonian symptoms, inducing side effects, or reaching the safety limit (5 mA). Contacts that had a wider therapeutic window and improved motor outcome were selected as active contacts, after which, the stimulation was set at 0.8 mA, 60 μs, and 130 Hz, and the current was increased by 0.2–0.4 mA each day. The final cDBS was set at 2 (–) C (+), 130 Hz, 60 μs, and 2.2 mA in both hemispheres, and his levodopa equivalent daily dose was reduced from 950 mg to 550 mg (levodopa/carbidopa 400 mg, four times, pramipexole 1.5 mg). One month post-surgery, we re-evaluated his motor symptoms. The MDS-UPDRS motor score was 27 points during med-off/stim-off, and the LFP intensity values were 1.20 μVp and 1.72 μVp in the left and right STN, respectively (Figure 1b). The improvement observed during the off-state following surgery may be attributed to the micro lesioning effect compared to that before surgery. During the med-off /stim-on phase, the MDS-UPDRS score was 22 points while the LFP intensity was 1.24 μVp (left) and 1.00 μVp (right). After a few weeks, the patient was hospitalized with adjustments to the aDBS setting. Prior to the start of the aDBS session, we verified the presence of a significant beta peak (16.6 Hz), and initiated aDBS to establish effective stimulation parameters. We defined the stimulation threshold of aDBS as the stimulation range between the current that elicits the minimum detectable effect (minimum stimulation) on cardinal symptoms and the current that elicits side effects (maximum stimulation). The aDBS stimulation was finally set with the upper stimulation at 3.5 mA on both sides and the lower stimulations at 0.7 mA and 1.2 mA on the left and right sides, respectively. The strength of the beta oscillations that was detected at the minimum and maximum stimulations was set as “upper strength of LFP” and “lower strength of LFP,” respectively. If LFP reached the upper strength of LFP, stimulation was gradually increased toward the maximum stimulation. If LFP reached the lower strength of LFP, stimulation was gradually decreased toward the minimum stimulation. The stimulation frequency and duration were set at 130 Hz and 60 μs, respectively. The patient returned to work the day following discharge. During the aDBS/outside the hospital phase, the MDS-UPDRS dyskinesia score was 0 points and 0 points during the cDBS/outside the hospital phase. The motor fluctuation score was 0 points during the aDBS phase/outside hospital phase and 3 points during the cDBS/outside the hospital phase. During the cDBS mode (outside the hospital), the mean strength of the beta band oscillation and stimulation were 291 least significant bits (LSB) and 2.2 mA, respectively. During the aDBS mode, the mean strength of the beta band oscillation and stimulation amplitude were less evident in the hospital setting (mean strength of beta oscillation: 144.6 LSB; mean amplitude of stimulation: 1.61 mA) than in the non-hospital setting (mean strength of beta oscillation: 280 LSB; mean amplitude of stimulation: 2.93 mA). The percentage exceeding the beta upper threshold was 12%, that below the threshold was 41%, and that between the threshold was 47% inside the hospital setting. Conversely, the percentage exceeding the beta upper threshold was 73%, that below the threshold was 15%, and that between the threshold was 12% in the non-hospital setting, while the stimulation successfully adapted to the fluctuations of LFP during the aDBS phase (Figure 2). The clinical course is summarized in Figure 3.

## Discussion

Previous reports have demonstrated the feasibility of aDBS in comparison to cDBS, which continuously delivers electrical stimulation regardless of beta band oscillation fluctuations (Little et al., 2016; Arlotti et al., 2018). Previous reports have also illustrated the effectiveness of aDBS over shorter periods (Little et al., 2013; Rosa et al., 2015; Gilron et al., 2021). To the best of our knowledge, this is the first report to demonstrate that the power or fluctuation of the beta band oscillation depends on the long-term living conditions. Furthermore, beta power and stimulation amplitude were higher outside the hospital than inside, suggesting that the aDBS successfully adapted its stimulation strength based on the beta power. In our case, beta band oscillations fluctuated more while the strength of the oscillation was higher outside the hospital than inside. A high beta band oscillation strength in the STN of patients with PD is correlated with the severity of motor symptoms, especially rigidity and bradykinesia (Kuhn et al., 2006). The strength of the beta band oscillation is influenced not only by movement (Feingold et al., 2015) or dopaminergic drugs (Weinberger et al., 2006), but also by several other factors (Urrestarazu et al., 2009; Duprez et al., 2019; Benis et al., 2020). A previous study has suggested that PD motor symptoms worsened when patients were subjected to stress (van der Heide et al., 2021), while training and relaxation programs mitigated motor symptoms of PD (Reuter et al., 2011). These factors would therefore differ between in-hospital and non-hospital conditions, and may attribute to the variation in the power of beta oscillations, which ultimately affects the fluctuation and mean strength of the stimulation amplitude. No reports have demonstrated the change in beta band oscillation throughout the day and the adaptation of DBS to the change in power of the beta band oscillation. The LFP may provide important information regarding the patient's daily condition, which may serve as a biomarker of PD.

In conclusion, these findings provide new insights into the development of new generation implantable aDBS devices for the treatment of PD.

## Data Availability Statement

All datasets presented in this study are included in the article.

## Ethics Statement

The patient gave written informed consent for the publication of any potentially identifiable images or data included in this article.

## Author Contributions

AN and YS made substantial contributions to the study concept and design, acquisition of the data, and manuscript for intellectual content. AF, JT, MH, HI, AU, and NH participated in drafting the article or critically revising it for important intellectual content. All authors gave final approval of the version to be submitted and any revised version.

## Conflict of Interest

The authors declare that the research was conducted in the absence of any commercial or financial relationships that could be construed as a potential conflict of interest.

## Publisher's Note

All claims expressed in this article are solely those of the authors and do not necessarily represent those of their affiliated organizations, or those of the publisher, the editors and the reviewers. Any product that may be evaluated in this article, or claim that may be made by its manufacturer, is not guaranteed or endorsed by the publisher.
